# Impact of the transcription factor nuclear factor 1 B T>C polymorphism on clozapine metabolism in vivo and expression of intestinal transporters in vitro

**DOI:** 10.1016/j.dmd.2025.100100

**Published:** 2025-05-20

**Authors:** Maria Solbakk, Robert Løvsletten Smith, Birgit M. Wollmann, Line Skute Bråten, Inger Johansson, Magnus Ingelman-Sundberg, Ole A. Andreassen, Espen Molden

**Affiliations:** 1 Center for Psychopharmacology, Division for Mental Health and Substance Abuse, Diakonhjemmet Hospital, Oslo, Norway; 2 Section for Pharmacology and Pharmaceutical Biosciences, Department of Pharmacy, University of Oslo, Oslo, Norway; 3 Section of Pharmacogenetics, Department of Physiology and Pharmacology, Karolinska Institute, Stockholm, Sweden; 4 Division of Mental Health and Addiction, NORMENT Centre, Oslo University Hospital, Oslo, Norway

**Keywords:** Caco-2 cells, Efflux-transporters, Pharmacogenomics, rs28379954, Therapeutic drug monitoring

## Abstract

Clozapine’s (CLZ) pharmacokinetics involves multiple enzymes and transporters, which may be influenced by genetic variability. A variant in the nuclear factor I B (NFIB) gene (rs28379954 T>C) has been associated with reduced CLZ serum concentrations. This study explored CLZ metabolism in relation to *NFIB* genotype in patients with known smoking habits. NFIB’s role in regulating gene expression of transporters relevant for intestinal absorption of CLZ was investigated using Caco-2/TC7 cells. Metabolite spectra of CLZ-treated patients (*n* = 285) were included from a therapeutic drug monitoring service. Formation of 30 CLZ metabolites was compared between patients carrying *NFIB CT* and *TT* diplotypes. To investigate *NFIB*’s possible role in regulating the expression of drug transporters of relevance for CLZ efflux (*ABCB1*, *ABCC1*, and *ABCG2*) or uptake (*SLC* transporter*s*), NFIB was overexpressed in Caco-2/TC7 cells. CLZ dose-adjusted concentration was 25% lower in *NFIB CT* versus *TT* carriers (*P* = .017). No significant differences in primary metabolites were found, but a secondary metabolite, N-desmethylclozapine cysteinyl, was increased by 1.89-fold in smoking *CT* versus *TT* carriers (*P* = .038). In Caco-2/TC7 cells, NFIB overexpression significantly suppressed the expression of *ABCB1* and *ABCG2* by 25%–30%. In summary, *NFIB CT* carriers require higher CLZ doses for optimal clinical effect, yet their metabolite profiles are similar to those of *TT* carriers, suggesting no differences in enzyme activity. Instead, Caco-2/TC7 experiments showed reduced *ABCB1* and *ABCG2* expression in NFIB-transfected cells. This may indicate that lower CLZ levels in *CT* carriers result from decreased NFIB-mediated inhibition of transporter expression. However, further in vivo studies are needed to clarify NFIB’s role in CLZ transport mechanisms.

**Significance Statement:**

This study shows that the *NFIB* rs28379954 *T>C* variant causes reduced clozapine serum levels and hence increased dose requirements to reach therapeutic levels for optimal clinical response. However, this effect seems to be independent of metabolic changes, suggesting alternative pharmacokinetic mechanisms at play. In vitro experiments further indicate that NFIB may regulate the expression of intestinal efflux transporters. Both findings provide a future foundation for genotype-guided dosing of clozapine in patients suffering from treatment-resistant schizophrenia.

## 1 Introduction

Clozapine (CLZ) is the most effective antipsychotic drug in the treatment of schizophrenia.^1^ Treatment with CLZ reduces the risk of suicide, discontinuation, and psychiatric hospitalizations and has shown superior effects over other antipsychotics in improving symptom scores and overall quality of life.^2^^,^^3^ Despite the documented benefits, its use in clinical practice is restricted to patients with treatment-resistant schizophrenia due to the risk of hematological toxicity, including the potentially fatal condition of agranulocytosis.^4^^,^^5^

The underlying mechanism of CLZ-induced agranulocytosis remains poorly understood but appears to involve an immunological response independent of dose/systemic exposure.^6^ In contrast, other adverse reactions, such as sedation, hypersalivation, and tonic-clonic seizures, are known to occur in a dose-dependent manner.^7^ Thus, the use of therapeutic drug monitoring (TDM) is important for managing these side effects, especially given the extensive variability in CLZ serum levels observed in patients receiving similar doses.^8^ Among the factors with the highest impact are smoking habits, age, sex, and comedication with interacting drugs.^8^^,^^9^ In sum, these factors account for about 50% of the observed interindividual variability in CLZ serum concentration*,* with tobacco smoking being the most significant contributor by reducing the serum levels by 30%–40%.^10^^,^^11^

The hepatic biotransformation of CLZ is complex, with CYP1A2 and CYP3A4 as the primary enzymes responsible for the formation of its 2 major metabolites: *N*-desmethylclozapine (DCL) and clozapine-*N*-oxide.^12^^,^^13^ These metabolites and CLZ per se are further biotransformed into a range of different metabolites.^14^^,^^15^ CLZ is also converted into reactive nitrenium ions, involving myeloperoxidases and possibly CYP3A4 and CYP2D6.^13^^,^^14^ These ions may contribute to CLZ’s hematological toxicity by inactivating granulocytes or triggering immune responses,^6^^,^^16^ but their formations are probably not important for the overall CLZ exposure.

There is some evidence indicating that CLZ may interact with various drug transporters, such as adenosine triphosphate-binding cassette membrane transporter B1 (ABCB1) (P-glycoprotein) and ABCG2 (breast cancer resistance protein).^12^ Consequently, variability in CLZ pharmacokinetics could be influenced by differences in both its metabolism and transport, which are processes that may be determined by many factors, including pharmacogenetic variability. Genetic variation has been described as an important factor for interindividual variation in CLZ levels.^12^ This includes pharmacogenomics of CYP1A-mediated metabolism^12^^,^^17^ and potentially ABCG2-mediated transport as well.^18^ A genome-wide association study also identified a novel rs28379954 *T>C* polymorphism in the gene encoding for nuclear factor 1 B (NFIB) of importance for CLZ levels.^19^

NFIB is a transcription factor involved in the regulation of gene expression during development and differentiation in several tissues, including the brain, liver, lungs, and kidneys.^20^^,^^21^ Its activity has been linked to both disease processes, such as certain cancers^20^^,^^21^ and pharmacogenomic effects, including increased metabolism of risperidone.^22^ In the context of CLZ, the genome-wide association study revealed that the minor *NFIB C* variant (∼10% frequency) was significantly associated with reduced CLZ levels when adjusting for smoking status.^19^ However, it is unclear whether the reduced CLZ levels in *NFIB C* carriers are related to increased metabolism or increased efflux in the intestinal wall.

Because the specific mechanisms by which the *NFIB* genotype is related to reduced CLZ levels remain unknown, the aim of the present study was to explore the impact of *NFIB* genotype on the formation of 30 CLZ metabolites in a large patient sample with known tobacco smoking habits, as well as to assess NFIB’s effect on the expression of genes encoding for CLZ transporters in colon carcinoma Caco-2/TC7 cells.

## 2 Materials and methods

### 2.1 Study design and patient inclusion

The CLZ patients were retrospectively included from the TDM/genotyping service at the Center for Psychopharmacology, Diakonhjemmet Hospital, in the timeframe of January 2019–October 2023. Patients who (1) had DNA samples biobanked for additional genetic analysis, (2) performed TDM of CLZ during the timeframe of the study period, and (3) had information about prescribed daily dose and blood sampling time on their requisition form, were considered for inclusion.

Patients were excluded if they met any of the following criteria: (1) age was <18 or >64 years, (2) serum levels of CLZ and DCL were <50 nmol/L (lower quantification limit of the analytical assay), (3) prescribed daily dose was outside the linear dose-concentration range of 100–900 mg, (4) blood samples were not collected within 10–30 hours postdose, (5) use of CLZ-interacting drugs (ie, carbamazepine, phenobarbitone, phenytoin, valproate, or fluvoxamine), and (6) recent smoking habits were not reported on the TDM requisition form. Some of the patients had multiple samples fulfilling the criteria for inclusion during the study period. In such cases, 3 measurements per patient were included by random computer-based selection.

The use of historical laboratory data in this study was approved by the Regional Committee for Medical and Health Research Ethics (REC#9393) and the Hospital Investigational Review Board without the requirement of informed consent because the use of retrospective data and rest blood samples was considered not to pose any risk or burden for the patients. All processing of personal and sensitive data was carried out anonymously.

### 2.2 NFIB genotyping

*NFIB* genotyping was carried out by extracting DNA from biobanked blood samples followed by targeted TaqMan-based analysis of rs28379954 *T>C*, as described previously.^23^ Briefly, the DNA extraction was accomplished from EDTA blood and performed by using the MagNA pure 24 Total NA Isolation kit I (Roche Diagnostics GmbH) on a MagNA Pure 24 instrument (Roche Diagnostics GmbH). A predesigned TaqMan-based real-time polymerase chain reaction assay *NFIB* rs28379954 *T>C* (Thermo Fisher Scientific) was used for the determination of *NFIB* genotype. The polymerase chain reaction reactions were run on a QuantStudio 12K Flex Real-Time polymerase chain reaction System (Thermo Fisher Scientific).

### 2.3 Metabolic profiling of CLZ

The method for CLZ and metabolite detection and quantification applied in the TDM routine has previoulsy been described elsewhere.^24^, ^25^, ^26^, ^27^ In short, all samples were prepared by protein precipitation in a semiautomated method using a Microlab Star pipetting robot (Hamilton) and were analyzed using a vanquish ultrahigh-performance liquid chromatography system (Thermo Fisher Scientific). Chromatographic separation was obtained using an XBridge BEH C18-column (2 *μ*m, 2.1 × 75 mm; Waters) with a gradient elution consisting of ammonium acetate buffer (pH = 4.8) and acetonitrile (20%–52%) at 35 °C. The ultrahigh-performance liquid chromatography system was coupled with a high-resolution mass spectrometer equipped with an Orbitrap detector set to positive ionization mode, scanning data at a resolution of 70,000 across a mass range of 100–1500 Da, allowing precise mass separation up to the fourth decimal. Quantification of CLZ and DCL was conducted using a standard calibration curve, with a lower detection limit of 20 nmol/L for both analytes. The validation parameters in the routine analysis at Center for Psychopharmacology allowed <5% inaccuracy.

Although information on serum concentration measurements of CLZ and DCL was obtained directly from the laboratory data system, metabolite profiles of CLZ were collected by reprocessing of the full-scan high-resolution mass spectrometer files from previously analyzed TDM samples. Levels of the remaining metabolites were quantified or semiquantified by retrospective reprocessing using the software TraceFinder 5.1 (Thermo Fisher Scientific). CLZ *N*-oxide, CLZ-*5N*-glucuronide, and CLZ *N+*-glucuronide concentrations were obtained by preparing standard curves of commercially purchased reference substances applied for retrospective reprocessing and quantification based on historical mass spectra. For the remaining 26 metabolites, reference standards were not available, so identification and quantification were based on accurate mass (with a mass tolerance of 5 ppm for the protonated molecular ion), isotope ratio assessment, and evaluation of the MS/MS spectra consistent with chemical structures of CLZ-derived compounds. Semiquantification of these latter metabolites was determined in arbitrary units based on chromatographic peak intensities with detector responses assumed to be linear and similar to that of CLZ. The metabolite-to-CLZ levels (metabolic ratios [MRs]) were used as a measure of formation for all metabolites.

### 2.4 Outcomes and statistics related to NFIB genotype in vivo

When assessing the impact of *NFIB* genotype on the formation of the various metabolites in the complex biotransformation profile of CLZ, mixed model analysis was conducted, with age, sex, and sampling time as covariates. Mixed model analysis allows for the inclusion of multiple samples per individual, hence taking into account intraindividual variability when estimating effects of fixed variables. The main outcome variables were dose-adjusted serum concentration (C/D ratio) of CLZ and MRs of the various metabolites in relation to *NFIB* genotype. C/D ratio ([nmol/L]/[mg/day]) is an indirect measure of clearance in vivo, whereas MR (metabolite-to-CLZ ratio) indicates the rate of metabolism exclusive to a specific pathway. To maintain consistency and facilitate comparisons between the genotype groups, all values were reported with 3 significant digits. This approach enables assessment of secondary metabolites with low peak intensities to provide comparable values between the *NFIB* genotype subgroups; however, it does not indicate the accuracy of the arbitrary units used. The C/D ratios and MRs were normalized prior to the statistical analysis using ln-transformation to ensure normal distribution of the outcome variables.

Considering the large impact smoking has on CLZ metabolism, the study population was stratified based on the patients’ smoking habits in the mixed model analyses. Information regarding smoking status was obtained by reviewing the respective TDM requisition forms, where “smoking” or “nonsmoking” is usually indicated. In cases where data on smoking status were missing, nonsmoker status was confirmed by the absence of the nicotine metabolite, cotinine, in the sample, assessed by reprocessing the associated full-scan high-resolution mass spectrometer files. However, cotinine levels alone were not used to define smokers because it is the polycyclic hydrocarbons in cigarette smoke, rather than nicotine, that are responsible for the effect smoking has on CLZ metabolism. Thus, the use of other nicotine-containing products could result in misclassification.

For comparisons of the demographic characteristics between *NFIB* genotype subgroups, information from the latest TDM sample/requisition available for the respective patient was applied. Statistical analyses were performed using Student’s *t* test for continuous variables and chi-squared test for dichotomous variables. The statistical analyses and graphic presentations were performed in R Studio (version 1.4.1103), and values of *P* < .05 were considered statistically significant.

### 2.5 Experiments in human colon carcinoma cell line Caco-2/TC7

Given the unknown potential impact of NFIB on drug transporter phenotypes, *in vitro* experiments were conducted using a transfected cell model to investigate NFIB’s effect on the expression of intestinal transporters known to be involved in CLZ efflux (*ABCB1*, *ABCC1*, and *ABCG2)* or influx (*SLC22A1*, *SLC22A2*, *SLC22A3*, and *SLCO1B1*). These experiments used colon carcinoma Caco-2/TC7 cells (a kind gift from Tommy B Andersson, AstraZeneca, Mölndal, Sweden) for *NFIB* transfection.

The Caco-2/TC7 cells were cultured in high glucose Dulbecco’s modified Eagle’s medium supplemented with 16.6% fetal bovine serum, 1 mM sodium pyruvate, 100 IU/mL penicillin, and 100 *μ*g/mL streptomycin. Cells were seeded onto 6-well plates and, once confluent, transfected with either 3 *μ*g of the pCMV3-NFIB expression plasmid (Sino Biological) or 3 *μ*g of an empty pCMV plasmid using Lipofectamine 3000 Transfection Reagent (Invitrogen). The cells were harvested after 48 h. RNA was isolated using QIAzol Lysis Reagent (Hilden), and cDNA synthesis was performed using Superscript III reverse transcriptase (Invitrogen). Gene expression was analyzed using TaqMan assays (Applied Biosystems) (Supplemental Table 1). TATA box binding protein was used as a housekeeping gene due to its stability and expression remaining unaffected by NFIB overexpression. Expression of drug transporter was compared between cells overexpressing NFIB and control cells in triplicates and statistically evaluated by Student’s *t* test.

## 3 Results

### 3.1 Demographics

A total of 4313 TDM measurements of CLZ and its metabolites were included from 582 previously genotyped patients, each with available data on daily dose and sampling time. Among these, 279 patients were excluded for not meeting the study’s criteria, with 157 patients excluded due to missing tobacco smoking status. The final analysis included 719 measurements from 285 patients. In the population, 23 patients (8.1% of the population) were identified as heterozygous carriers of the *NFIB C* variant allele (one patient with a homozygous *CC* genotype was excluded due to missing information on smoking status). The remaining 262 patients were homozygous carriers of the *TT* genotype. Table 1 provides a summary of the demographic characteristics of the included patients, with no significant differences observed in any of the listed variables between carriers and noncarriers of the *NFIB C* variant allele. Overall, 51.9% of the total population were smokers, indicating a high smoking prevalence. Among *CT* carriers, 56.5% reported smoking, compared to 51.5% of *TT* carriers.

**Table 1:** Population demographics

Variables	All patients	NFIB C/T genotype	NFIB T/T genotype	*P* value
Number of patients, *n* (measurements)	285 (719)	23 (60)	262 (659)	-
Female, *n* (%)	112 (39.3%)	10 (43.5%)	102 (38.9%)	.8372
Age, y; mean (95% CI)	39.55 (38.2, 40.9)	43.61 (37.5, 49.7)	39.19 (37.8, 40.6)	.2075
Daily dose, mg/day; mean (95% CI)	345.5 (326.2, 364.8)	394.6 (316.9, 472.2)	341.2 (321.3, 361.1)	.1404
Sampling time, h; mean (95% CI)	13.9 (13.6, 14.2)	13.3 (12.7, 13.9)	14.0 (13.7, 14.3)	.0741
Smokers, *n* (%)	148 (51.9%)	13 (56.5%)	135 (51.5%)	.8087
Absolute serum level CLZ; nmol/L; mean (95% CI)	1310.6 (1220.8, 1400.5)	1298.5 (923, 1674)	1311.7 (1218.9, 1404.5)	.8833
Absolute serum level DCL; nmol/L; mean (95% CI)	907.7 (851.3, 964)	834.5 (678.7, 990.4)	914.1 (854.1, 974)	.9078

The statistical analyses used the latest sample per patient. Chi-squared test was applied to the proportions (eg, gender, smoking status); Student’s *t* test was applied to the continuous variables (eg, age, daily dose, sampling time, and serum levels).

Of the 285 patients included, 112 (39%) also participated in our previous genome-wide association study,^19^ with an overlap of 13 *CT* carriers. In this study, we exclusively used new TDM measurements from overlapping patients to investigate an additional objective, that is, to examine the complete metabolite profiles of CLZ (30 metabolites) in relation to the *NFIB* genotype.

### 3.2 Effect of NFIB genotype on dose-adjusted CLZ concentration

In the mixed model analysis adjusted for age, sex, sampling time, and tobacco smoking status, the C/D ratio of CLZ in *NFIB CT* carriers was 25% lower than in *TT* carriers, regardless of smoking status (fold change: 0.75; confidence interval [CI]: 0.59–0.95; *P* = .0169; Table 2). Consistent with previous findings, the multivariate analysis revealed a significant effect of sex on CLZ levels, with women showing a 20% higher C/D ratio than men (fold change: 1.20; CI: 1.05–1.37; *P* < .01; Table 2). Additionally, the analysis showed a 51% higher C/D ratio estimate in nonsmoking compared to smoking patients (fold change: 1.51; CI: 1.33–1.73; *P* < .0001; Table 2).

**Table 2:** Effect of NFIB CT diplotype on dose-adjusted serum concentration of clozapine in smoking or nonsmoking patients adjusting for age, sex, and sampling time

C/D CLZ			
	Geometric mean (95% CI)	Fold change	*P* value
All patients			
CT	2.75 (2.19–3.45)	0.75 (0.59–0.95)	.0169∗
TT	3.67 (3.43–3.93)		
Female	3.48 (3.02–4.01)	1.2 (1.05–1.37)	.0081∗
Male	2.90 (2.55–3.30)		
Smokers	2.58 (2.26–2.95)	0.66 (0.579–0.753)	<.0001∗
Nonsmokers	3.91 (3.41–4.48)		
Smokers			
CT	2.15 (1.56–2.70)	0.714 (0.511–0.999)	**.**0495∗
TT	3.00 (2.70–3.34)		
Female	2.80 (2.26–3.45)	1.21 (0.988–1.49)	.0651
Male	2.31 (1.92–2.76)		
Nonsmokers			
CT	3.73 (2.67–5.22)	0.834 (0.589–1.18)	.3022
TT	4.48 (4.08–4.92)		
Female	4.51 (3.69–5.52)	1.22 (1.01–1.46)	.0349∗
Male	3.71 (3.06–4.49)		

∗ Indicates significance.

In the subpopulation analysis stratified by smoking habits, the difference in C/D ratio between *NFIB CT* and *TT* carriers remained significant only among smokers, with *CT* carriers showing a 28.6 % lower C/D ratio (fold change: 0.714; CI: 0.511–0.999; *P* = .0495; Table 2). Although the C/D ratio was also lower in *CT* carriers in nonsmoking patients, this difference was not statistically significant compared to *TT* carriers (fold change: 0.834; 95% CI: 0.589–1.18; *P* = .3022; Table 2).

### 3.3 Effect of NFIB genotype on metabolite formation

**Fig. 1:**
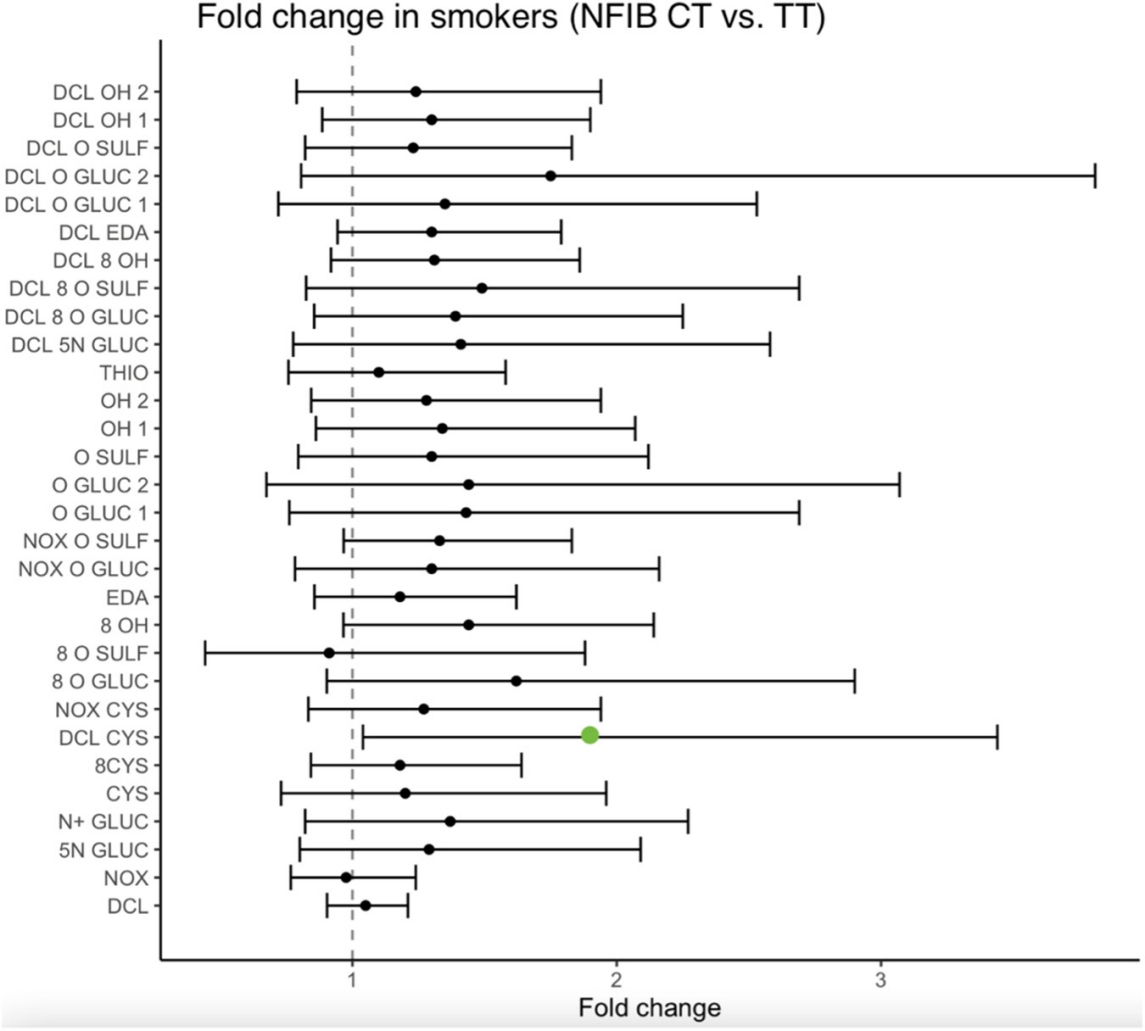
Effect of *NFIBrs28379954 T>C* diplotype on metabolic ratios in smoking patients adjusting for age, sex, and sampling time. The data are analyzed using linear mixed model. The figure displays the fold change (95% CIs) in *NFIB CT* carriers (heterozygous for the target allele) versus *NFIB TT* carriers (homozygous for the target allele). Significant effect was observed for the metabolic ratio of *N*-desmethylclozapine cysteine: DCL-CYS/DCL (fold change: 1.89; CI: 1.04–3.44; *P* = .0384). Supplemental Table 2 contains the effect estimates, fold changes, and 95% CIs of the rest of the metabolites. CI, confidence interval; DCL, *N*-desmethylclozapine; DCL-CYS/DCL, DCL-cysteinyl; NFIB, nuclear factor 1 B.

**Fig. 2:**
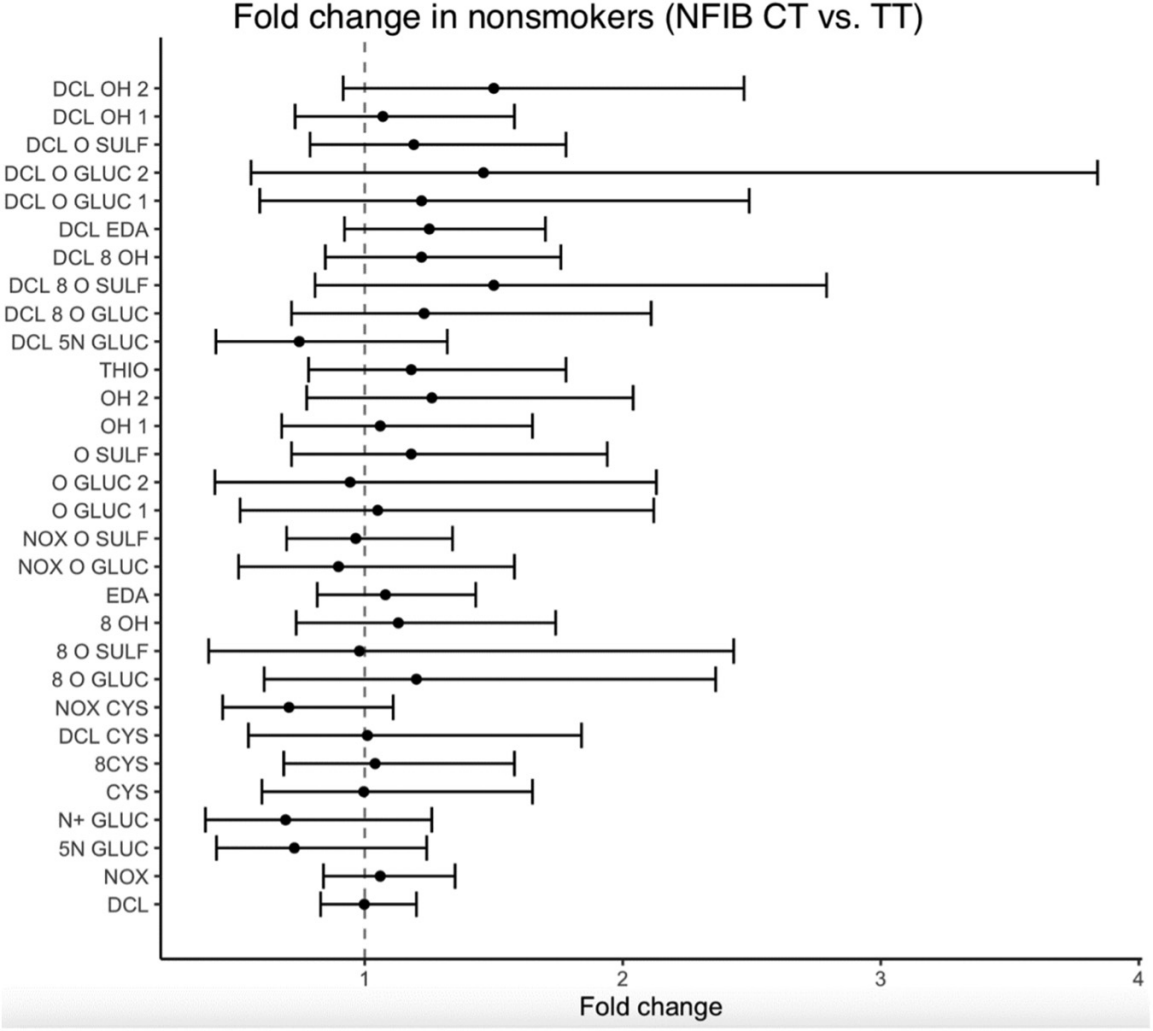
Effect of *NFIBrs28379954 T>C* diplotype on metabolic ratios in nonsmoking patients adjusting for age, sex, and sampling time. The data are analyzed using linear mixed model. The figure displays the fold change (95% CIs) in *NFIB CT* carriers (heterozygous for the target allele) versus *NFIB TT* carriers (homozygous for the target allele). No significant effect was observed for the metabolic ratios of the 30 metabolites. Supplemental Table 3 contains the effect estimates, fold changes, and 95% CIs of all the metabolites. CI, confidence interval; NFIB, nuclear factor 1 B.

The results from the stratified statistical analyses on the effects of *NFIB* genotype on the formation of 30 CLZ metabolites, adjusted for age, sex, and sampling time, are presented in Fig. 1 and Supplemental Table 2 for smokers and Fig. 2 and Supplemental Table 3 for nonsmokers. With the exception of one secondary metabolite—DCL-cysteinyl (DCL-CYS) —which showed significantly higher levels in smoking *CT* carriers (fold change: 1.89; CI: 1.04–3.44; *P* = .0384; Fig. 1), no significant differences in metabolic formation were observed between *CT* and *TT* carriers (Fig. 1). Although higher metabolite levels were generally noted in smoking *CT* carriers, none approached statistical significance compared to *TT* carriers (Fig. 1). In the nonsmoking group, there were no consistent trends in metabolic formation between genotype subgroups (Supplemental Table 3).

### 3.4 Effect of NFIB on expression of CLZ transporters in vitro

**Fig. 3:**
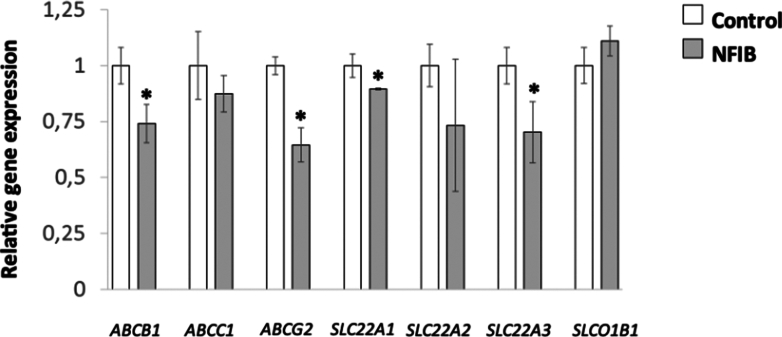
Effect of overexpression of NFIB on the expression of transport genes in Caco-2/TC7 cells. The figure shows the relative gene expression of transporters in Caco-2/TC7 cells transfected with pCMV3-NFIB plasmid compared to control Caco-2/TC7 cells transfected with empty pCMV3 plasmid. TATA box binding protein was used as a housekeeping gene. The data shown are from a representative experiment performed in triplicates. Statistics were performed using Student’s *t* test ∗*P* < .05; Genes and corresponding transporters: *ABCB1* – p-glycoprotein; *ABCC1* -MRP1; *ABCG2* – BCRP; *SLC22A2* – OCT2; *SLC22A3* – OCT3; *SLCO1B1 –* OATP1B1. NFIB, nuclear factor 1 B.

We also investigated whether NFIB affects the in vitro expression of intestinal transporters reported to be involved in CLZ efflux.^18^^,^^28^^,^^29^ To do this, we assessed the extent of NFIB regulation using colon carcinoma Caco-2/TC7 cells, a model previously employed to study CLZ’s intestinal transporters.^30^ The results revealed a 25%–30% reduction in the expression of *ABCB1* and *ABCG2*, which encode for P-glycoprotein and breast cancer resistance protein, respectively, in NFIB-overexpressed vs. control cells (*P* < .05; Fig. 3). The *SLC22A3* expression was also significantly reduced in the transfected cells; however, absolute expression in these cells was very low compared to the other transporters. There were no apparent changes in expression levels of *ABCC1, SLC22A1, SLC22A2,* or *SLCO1B1 mRNAs* in transfected versus control cells.

## 4 Discussion

In this study, we investigated whether the *NFIB* rs28379954 *T>C* polymorphism, which is associated with reduced CLZ serum concentration, affects the formation of CLZ metabolites. We hypothesized that *NFIB C* carriers exhibited increased metabolite formation compared to *TT* carriers, but no significant differences in primary metabolites were found between the subgroups. The only significant difference was a 1.89-fold increase in the MR of the secondary DCL-CYS metabolite in smoking *CT* carriers. Glutathione (cysteine) conjugation is considered a minor pathway in CLZ elimination.^13^^,^^31^ Therefore, it is unlikely that increased formation of DCL-CYS is the underlying cause of the 25% reduction in the CLZ concentration-to-dose (C/D) ratio observed in *NFIB CT* carriers. However, because DCL-CYS is derived from a reactive nitrenium metabolite, NFIB might still play a role in gene regulation related to CLZ toxicity.

Glutathione *S*-transferases are key enzymes responsible for detoxifying reactive nitrenium ions into glutathione metabolites,^14^ which are subsequently transformed into cysteinyl derivatives.^26^^,^^32^ The increased DCL-CYS formation in smoking *NFIB CT* carriers might therefore be due to increased glutathione *S*-transferase expression, providing more efficient inactivation of toxic nitrenium ions. However, an alternative explanation is that NFIB also regulates the expression of myeloperoxidases, which are known to catalyze the formation of nitrenium ions of CLZ. Combined with the higher oxidative stress generally observed in cigarette smokers,^33^^,^^34^ one may hypothesize that smoking *NFIB C* carriers are at greater risk of toxic side effects, such as neutropenia/agranulocytosis and myocarditis. However, these are speculations that need to be clarified by metabolic analyses of serum samples from patients who experience hematological and/or cardiological toxicity.

**Fig. 4:**
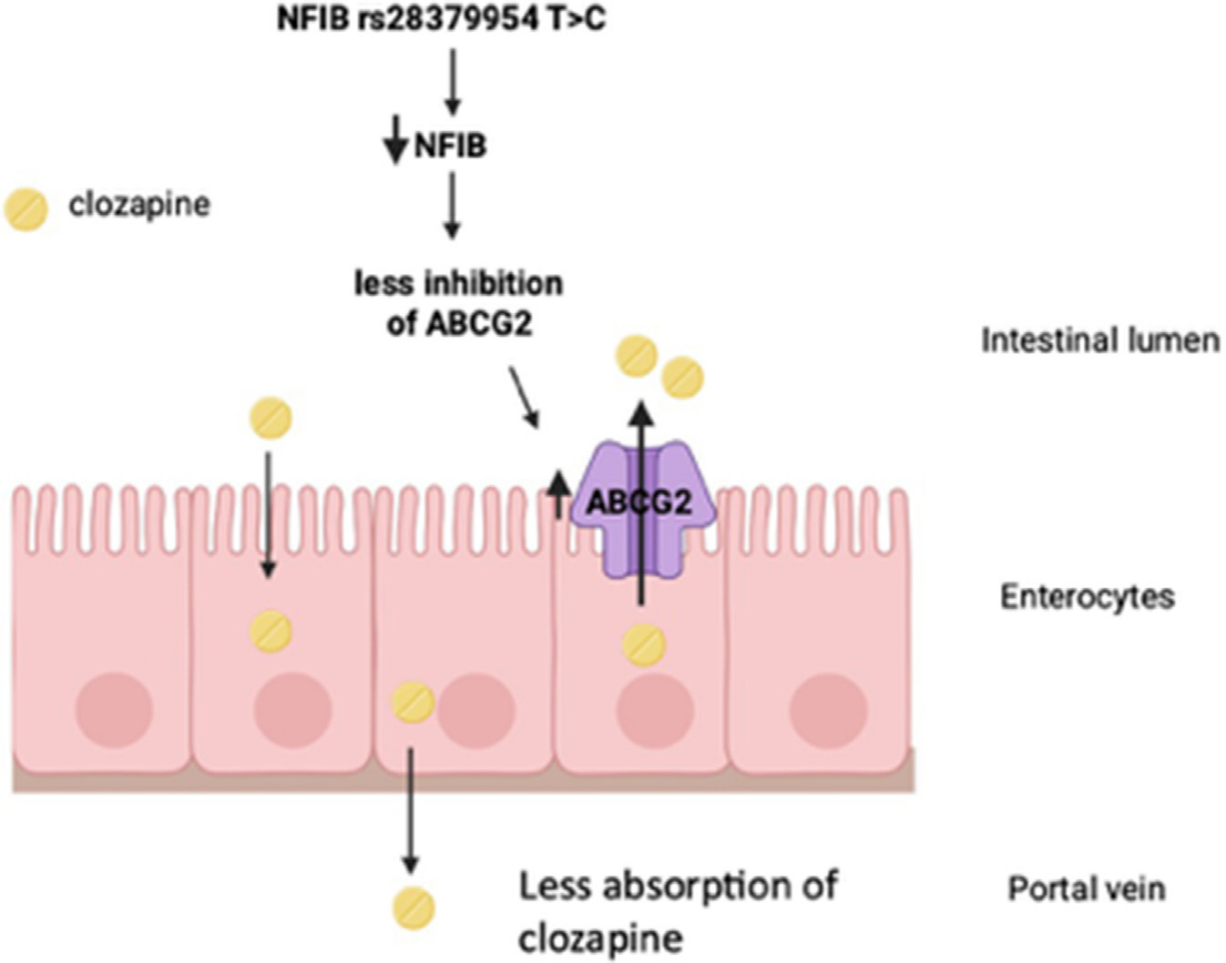
Proposed mechanism of the NFIB polymorphism−mediated influence on clozapine uptake. This figure illustrates a potential mechanism by which the *NFIB CT* genotype affects clozapine uptake based on the experiments in the Caco-2 cells (Fig. 3), similar to its regulation of CYP2D6-catalyzed risperidone metabolism as described by Lenk et al.^31^ In individuals carrying the *NFIB CT* genetic variant, the expression of NFIB is reduced causing a decreased suppression of the ABCG2 transporter expression and reduced uptake of clozapine into the portal vein (Figure made by Biorender).

The significant 25% reduction in the CLZ C/D ratio in *CT* carriers is consistent with the findings from our previous study, which reported a 40% reduction.^19^ To explore alternative mechanisms for the reduced CLZ levels in patients with the *NFIB C* variant, we analyzed the potential effects of NFIB on the expression of transporters associated with CLZ disposition. Overexpression of NFIB in colon carcinoma Caco-2/TC7 cells caused significant NFIB-dependent inhibition of *ABCB1* and *ABCG2* gene expression (Fig. 3). Previous research has demonstrated that patients with the *NFIB CT* diplotype exhibit reduced NFIB protein expression in hepatocyte cell nuclei.^22^ This reduction leads to diminished NFIB-dependent inhibition of *CYP2D6* expression, resulting in an increased rate of risperidone hydroxylation. Drawing an analogy with the NFIB-CYP2D6 interactions and the current findings, we hypothesize that the decreased CLZ serum levels observed in carriers of the *NFIB CT* diplotype are attributable to reduced NFIB-mediated suppression of intestinal *ABCG2* and/or *ABCB1* expression. This downregulated suppression of transporter gene transcription might increase the expression of *ABCG2*/*ABCB1* in enterocytes and potentially cause increased CLZ efflux into the intestinal lumen, subsequently lowering its systemic levels and oral bioavailability (Fig. 4).^31^

These mechanistic insights align with the obtained results and could serve as the basis for future in vivo studies investigating the hypothesis that pharmacogenetics of intestinal efflux transporters influence CLZ serum concentrations. A few studies with limited patient population have investigated the relationship between CLZ and *ABCB1* (P-glycoprotein), reporting conflicting results.^28^^,^^29^^,^^35^ For *ABCG2*, a single study suggests an association between a variant in the *ABCG2* gene and elevated C/D ratio of CLZ,^18^ suggesting its potential significance in CLZ transport. Interestingly, 2 studies have indicated that smoking upregulates *ABCG2* expression at both mRNA and protein levels, potentially amplifying the impact of the *NFIB CT* polymorphism in smokers.^36^^,^^37^ However, further investigations are needed to explore whether protein levels of drug-metabolizing enzymes and/or drug transporters in human hepatic and intestinal biopsies are determined by *NFIB* genotype.

Because of the naturalistic design and incomplete information on nongenetic factors affecting CLZ levels, the current study faces some limitations. Such factors include various access to potentially interacting comedications and the degree of adherence to treatment. Although patients identified with interacting comedications written on the requisition form, for example, known enzyme-interacting drugs, were excluded, we cannot rule out the use of additional agents interacting with CLZ pharmacokinetics. Typically, adherence rates for CLZ are high due to the frequent TDM and hematological assessments required.^8^ However, although we excluded cases of complete nonadherence by removing patients with undetectable CLZ concentrations, we cannot guarantee that all included patients consistently followed their prescribed treatment regimen, which could influence our findings. We also lacked reference standards for several of the CLZ metabolites, which necessitated the use of peak intensities as a proxy for metabolite formation. This approach assumes similar detector responses to that of CLZ, which may not be true for all compounds. Additionally, we encountered small peak intensities in many of the secondary metabolites, resulting in low MRs. To facilitate the comparison between NFIB genotype groups, we consistently used 3 valid digits. Although this approach aids consistency and ability to compare metabolite formations in carriers and noncarriers of the *NFIB C* variant, it does not reflect the accuracy of the semiquantified metabolite levels. Despite these constraints, the study’s strengths include a substantial sample size of 285 patients with multiple samples from the TDM data base, providing a unique real-world dataset. Additionally, the recorded tobacco smoking habits of patients, a crucial factor due to its known impact on CLZ metabolism, further enhance the study’s reliability.

In conclusion, this study demonstrates that patients carrying the minor *NFIB CT* diplotype are at an increased risk for subtherapeutic serum concentrations, a concern that is especially relevant among smokers. The lack of *NFIB CT*-related changes in the formation of 29 out of 30 CLZ metabolites suggests that the reduced serum level is not due to increased CLZ metabolism. Instead, experiments in Caco-2/TC7 cells indicate that the effect of *NFIB CT* in lowering CLZ levels could arise from reduced NFIB-mediated suppression of *ABCG2* and/or *ABCB1* expression. However, further research is necessary to establish the mechanisms of NFIB-mediated regulation of CLZ intestinal transport, as such insights could advance personalized treatment strategies for patients undergoing CLZ therapy.

## Conflict of interest

Maria Solbakk reports financial support was provided by South-Eastern Norway Regional Health Authority. Ole A. Andreassen reports a relationship with Cortechs.ai and Precision-health.ai that includes: consulting or advisory. Ole A. Andreassen reports a relationship with Lundbeck that includes: speaking and lecture fees. Ole A. Andreassen reports a relationship with Janssen Pharmaceuticals, Inc, that includes: speaking and lecture fees. Ole A. Andreassen reports a relationship with Sunovion that includes: speaking and lecture fees. Ole A. Andreassen reports a relationship with Otsuka Pharma Scandinavia AB that includes: speaking and lecture fees. Espen Molden reports a relationship with Lundbeck that includes: speaking and lecture fees. Espen Molden reports a relationship with Otsuka Pharma Scandinavia AB that includes: speaking and lecture fees. If there are other authors, they declare that they have no known competing financial interests or personal relationships that could have appeared to influence the work reported in this paper.
