# Supplementary material for: Impact of the transcription factor nuclear factor 1 B T>C polymorphism on clozapine metabolism in vivo and expression of intestinal transporters in vitro
Source: Drug Metab Dispos. 2025 May 20;53(7):100100. doi: 10.1016/j.dmd.2025.100100 (PMC12405907; doi:10.1016/j.dmd.2025.100100)
Supplement: Supplementary Tables 1-3 [file mmc1.docx]

**Supplemental Table 1.** TaqMan Assays (from Applied Bio-systems, Waltham, MA, USA) used for gene expression analysis.

| **Gene** | **Taqman Assay** |
| --- | --- |
| ***TBP***  (housekeeping gene) | Hs00427620_m1 |
| ***ABCB1*** | Hs00184500_m1 |
| ***ABCC1*** | Hs01561502_m1 |
| ***ABCG2*** | Hs01053790_m1 |
| ***SLC22A1*** | Hs00427552_m1 |
| ***SLC22A2*** | Hs00533907_m1 |
| ***SLC22A3*** | Hs00222691_m1 |
| ***SLCO1B1*** | Hs00272374_m1 |

**Supplemental Table 2.** Metabolic ratios of 30 clozapine metabolites for smoking NFIB CT variant and TT wildtype carriers using linear mixed model adjusting for age, sex and sampling time.

|  | **Smoker CT** | **Smoker TT** | **Fold change** | **p-value** |
| --- | --- | --- | --- | --- |
| **MR DCL** | 0.758 (0.661-0.870) | 0.725 (0.692-0-759) | 1.05 (0.904-1.21) | 0.5461 |
| **MR NOX** | 0.103 (0.0823-0.130) | 0.106 (0.0982-0.114) | 0.976 (0.767-1.24) | 0.8447 |
| **MR 5N GLUC** | 0.0745 (0.0472-0.118) | 0.0576 (0.0495-0.067) | 1.29 (0.801-2.09) | 0.2905 |
| **MR N+ GLUC** | 0.0878 (0.0541-0.1425) | 0.0643 (0.0548-0.0754) | 1.37 (0.821-2.27) | 0.2276 |
| **MR CYS** | 0.000184 (0.000115-0.000293) | 0.000154 (0.000132-0.000178) | 1.2 (0.73-1.96) | 0.4742 |
| **MR 8 CYS** | 0.00448 (0.00327-0.00614) | 0.00381 (0.00343-0.00423) | 1.18 (0.843-1.64) | 0.3373 |
| **MR DCL CYS** | 1.80e-4 (1.02e-4-0.000318) | 9.53e-5 (7.84e-5-0.000116) | 1.89 (1.04-3.44) | **0.0384** |
| **MR NOX CYS** | 0.0105 (0.00702-0.01565) | 0.00825 (0.00726-0.00939) | 1.27 (0.833-1.94) | 0.2639 |
| **MR CLZ 8 O GLUC** | 0.00155 (0.000890-0.00269) | 0.000957 (0.000793-0.00115) | 1.62 (0.903-2.9) | 0.1053 |
| **MR CLZ 8 O SULF** | 0.000104 (5.34e-5 – 0.000204) | 0.000114 (9.01e-5 – 0.000145) | 0.912 (0.443-1.88) | 0.8008 |
| **MR CLZ 8 OH** | 0.0899 (0.0616-0.131) | 0.0626 (0.0552-0.071) | 1.44 (0.966-2.14) | 0.0736 |
| **MR CLZ EDA** | 0.00214 (0.00158-0.00289) | 0.00182 (0.00164-0.00201) | 1.18 (0.856-1.62) | 0.3142 |
| **MR NOX O GLUC** | 0.0135 (0.00835-0.0218) | 0.0104 (0.00883-0.0122) | 1.3 (0.783-2.16) | 0.3094 |
| **MR NOX O SULF** | 0.0965 (0.0714-0.1306) | 0.0726 (0.0656-0.0803) | 1.33 (0.967-1.83) | 0.0789 |
| **MR CLZ O GLUC 1** | 0.000265 (0.000146-0.000482) | 0.000185 (0.000150-0.000229) | 1.43 (0.761-2.69) | 0.2642 |
| **MR CLZ O GLUC 2** | 0.000299 (0.000146-0.000612) | 0.000208 (0.000163-0.000265) | 1.44 (0.675-3.07) | 0.3440 |
| **MR CLZ O SULF** | 0.00358 (0.00225-0.00570) | 0.00276 (0.00236-0.00322) | 1.3 (0.795-2.12) | 0.2949 |
| **MR CLZ OH 1** | 0.00958 (0.00632-0.01453) | 0.00717 (0.00624-0.00824) | 1.34 (0.862-2.07) | 0.1933 |
| **MR CLZ OH 2** | 0.0496 (0.0334-0.0443) | 0.0388 (0.0340-0.0443) | 1.28 (0.844-1.94) | 0.2446 |
| **MR CLZ THIOMETHYL** | 0.0923 (0.0651-0.1308) | 0.0842 (0.0750-0.0947) | 1.1 (0.758-1.58) | 0.6260 |
| **MR DCL 5N GLUC** | 0.00223 (0.00126-0.00394) | 0.00157 (0.00130-0.00190) | 1.41 (0.776-2.58) | 0.2561 |
| **MR DCL 8 O GLUC** | 0.0123 (0.00775-0.0194) | 0.00884 (0.00758-0.0103) | 1.39 (0.855-2.25) | 0.1833 |
| **MR DCL 8 O SULF** | 0.000684 (0.000392-0.001194) | 0.000460 (0.000379-0.000557) | 1.49 (0.825-2.69) | 0.1843 |
| **MR DCL 8 OH** | 1.35 (0.965-1.88) | 1.03 (0.922-1.15) | 1.31 (0.919-1.86) | 0.1356 |
| **MR DCL EDA** | 0.0178 (0.0132-0.0241) | 0.0137 (0.0124-0.0151) | 1.3 (0.944-1.79) | 0.1070 |
| **MR DCL O GLUC 1** | 0.00150 (0.000831-0.00273) | 0.00112 (0.000908-0.00137) | 1.35 (0.72-2.53) | 0.3474 |
| **MR DCL O GLUC 2** | 0.000707 (0.000339-0.001476) | 0.000404 (0.000315-0.000517) | 1.75 (0.806-3.81) | 0.1552 |
| **MR DCL O SULF** | 0.0218 (0.0149-0.0319) | 0.0178 (0.0157-0.0202) | 1.23 (0.821-1.83) | 0.3176 |
| **MR DCL OH 1** | 0.0611 (0.0425-0.0878) | 0.0471 (0.0417-0.0531) | 1.3 (0.886-1.9) | 0.1797 |
| **MR DCL OH 2** | 0.0162 (0.0106-0.0248) | 0.0131 (0.0114-0.0150) | 1.24 (0.789-1.94) | 0.3508 |

**Supplemental Table 3.** Metabolic ratios of 30 clozapine metabolites for nonsmoking NFIB CT variant and TT wildtype carriers using linear mixed model adjusting for age, sex and sampling time.

|  | **Nonsmoker CT** | **Nonsmoker TT** | **Fold change** | **p-value** |
| --- | --- | --- | --- | --- |
| **MR DCL** | 0.661 (0.553-0.790) | 0.662 (0.629-0.696) | 0.998 (0.829-1.2) | 0.9862 |
| **MR NOX** | 0.0830 (0.0661-0.1042) | 0.0779 (0.0730-0.0832) | 1.06 (0.84-1.35) | 0.6002 |
| **MR 5N GLUC** | 0.0437 (0.0261-0.0732) | 0.0602 (0.0519-0.0697) | 0.727 (0.425-1.24) | 0.2408 |
| **MR N+ GLUC** | 0.0411 (0.0232-0.0728) | 0.0593 (0.0504-0.0698) | 0.693 (0.382-1.26) | 0.2247 |
| **MR CYS** | 0.000149 (9.19*e-5-0.000242) | 0.000150 (1.30e-4-0.000172) | 0.996 (0.601-1.65) | 0.9864 |
| **MR 8 CYS** | 0.00332 (0.00222-0.00495) | 0.00319 (0.00285-0.00358) | 1.04 (0.686-1.58) | 0.8546 |
| **MR DCL CYS** | 0.000119 (6.67e-5-0.000213) | 0.000119 (9.99e-5-0.000141) | 1.01 (0.549-1.84) | 0.9860 |
| **MR NOX CYS** | 0.00673 (0.00436-0.0104) | 0.00953 (0.00841-0.0108) | 0.706 (0.449-1.11) | 0.1304 |
| **MR CLZ 8 O GLUC** | 0.000689 (0.000360-0.001321) | 0.000574 (0.000477-0.000692) | 1.2 (0.61-2.36) | 0.5952 |
| **MR CLZ 8 O SULF** | 7.93e-5 (3.28e-5 – 0.000191) | 8.10e-5 (6.50e-5 – 0.000101) | 0.979 (0.395-2.43) | 0.9638 |
| **MR CLZ 8 OH** | 0.0427 (0.0282-0.0648) | 0.0378 (0.0336-0.0425) | 1.13 (0.734-1.74) | 0.5759 |
| **MR CLZ EDA** | 0.00122 (0.000931-0.00160) | 0.00113 (0.00104-0.00122) | 1.08 (0.816-1.43) | 0.5860 |
| **MR NOX O GLUC** | 0.00654 (0.00380-0.01124) | 0.00728 (0.00622-0.00852) | 0.898 (0.511-1.58) | 0.7062 |
| **MR NOX O SULF** | 0.0663 (0.0484-0.0907) | 0.0687 (0.0628-0.0751) | 0.965 (0.697-1.34) | 0.8311 |
| **MR CLZ O GLUC 1** | 0.000123 (6.24e-5 – 0.000241) | 0.000117 (9.54e-5 – 0.000144) | 1.05 (0.517-2.12) | 0.8988 |
| **MR CLZ O GLUC 2** | 0.000164 (7.52e-5 - 0.000359) | 0.000174 (1.39e-4 – 0.000218) | 0.943 (0.419-2.13) | 0.8874 |
| **MR CLZ O SULF** | 0.00239 (0.00148-0.00387) | 0.00203 (0.00177-0.00233) | 1.18 (0.716-1.94) | 0.5152 |
| **MR CLZ OH 1** | 0.00570 (0.00372-0.00873) | 0.00539 (0.00477-0.00608) | 1.06 (0.678-1.65) | 0.8038 |
| **MR CLZ OH 2** | 0.0327 (0.0206-0.0521) | 0.0261 (0.0229-0.0297) | 1.26 (0.775-2.04) | 0.3526 |
| **MR CLZ THIOMETHYL** | 0.0938 (0.0631-0.1394) | 0.0794 (0.0710-0.0889) | 1.18 (0.782-1.78) | 0.4260 |
| **MR DCL 5N GLUC** | 0.00142 (0.000825-0.00246) | 0.00191 (0.001633-0.00223) | 0.746 (0.423-1.32) | 0.3081 |
| **MR DCL 8 O GLUC** | 0.0070 (0.00417-0.01177) | 0.00570 (0.00492-0.00661) | 1.23 (0.716-2.11) | 0.4535 |
| **MR DCL 8 O SULF** | 0.000528 (0.000291-0.000958) | 0.000352 (0.000296-0.000418) | 1.5 (0.807-2.79) | 0.1976 |
| **MR DCL 8 OH** | 0.875 (0.616-1.245) | 0.717 (0.649-0.792) | 1.22 (0.847-1.76) | 0.2826 |
| **MR DCL EDA** | 0.0149 (0.0111-0.0200) | 0.0119 (0.0109-0.0129) | 1.25 (0.921-1.7) | 0.1494 |
| **MR DCL O GLUC 1** | 0.000825 (0.000414-0.001646) | 0.000679 (0.000557-0.000828) | 1.22 (0.593-2.49) | 0.5922 |
| **MR DCL O GLUC 2** | 0.000603 (0.000238-0.001528) | 0.000412 (0.000321-0.000527) | 1.46 (0.559-3.84) | 0.4344 |
| **MR DCL O SULF** | 0.0144 (0.00976-0.0214) | 0.0122 (0.0109-0.0136) | 1.19 (0.788-1.78) | 0.4117 |
| **MR DCL OH 1** | 0.0365 (0.0252-0.0529) | 0.0341 (0.0307-0.0378) | 1.07 (0.73-1.58) | 0.7195 |
| **MR DCL OH 2** | 0.01453 (0.00903-0.0234) | 0.00967 (0.00845-0.0111) | 1.5 (0.916-2.47) | 0.1063 |
